# Disentangling the Risks Associated With Weight Status, Diet, and Physical Activity

**Published:** 2009-09-15

**Authors:** Jan Warren-Findlow, Steven P. Hooker

**Affiliations:** Department of Public Health Sciences; University of South Carolina, Columbia, South Carolina

In 2005, the Centers for Disease Control and Prevention (CDC) estimated that 365,000 US deaths annually are attributable to poor diet and lack of physical activity (1). The methods used to calculate these deaths were based largely on body mass index (BMI) and, thus, obesity, instead of the 2 modifiable behaviors themselves. Poor diets (those that are high in calories, fat, and sugar) contribute to the prevalence of overweight and obesity; two-thirds of US adults are overweight or obese (2). National surveillance systems indicate that more than half of US adults do not meet the recommended level of moderate-intensity or vigorous-intensity physical activity, and physical activity levels decrease dramatically with age (3). Almost one-fourth of US adults report no leisure-time physical activity (4).

Together, poor diet and sedentary lifestyle assuredly contribute to a burgeoning obesity epidemic with corresponding increases in morbidity and mortality. Overweight and obese people are not, however, universally at risk for cardiovascular and metabolic diseases and their associated chronic disease risk. Approximately one-third of obese people do not manifest any clinical or subclinical risk factors for cardiovascular disease (CVD) such as dyslipidemia, hypertension, or elevated blood glucose (5). They documented that the strongest predictor for CVD risk is low levels of cardiorespiratory fitness (CRF).

Indeed, other studies indicate that normal-weight adults who are sedentary are at increased risk for CVD-related outcomes than are overweight or obese adults who are aerobically fit (6,7). Fitness, as assessed by a treadmill test, is also a protective factor for premature death in older adults regardless of weight status (8). Regular moderate-to-vigorous physical activity substantially improves CRF, and a higher level of CRF, as demonstrated in the Aerobics Center Longitudinal Study, is highly protective for stroke and other forms of CVD, regardless of other risk factors such as obesity (6,7,9).

This research demonstrates the potential flaw in using weight status as a surrogate for physical activity-related chronic disease risk. It seems to be poor science to simply use BMI to infer CVD risk, unless physical activity or CRF have been measured accurately and accounted for. Therefore, health researchers should take measures to disentangle the effects of diet and physical activity to understand the determinants and consequences of each relative to weight status and, more importantly, overall chronic disease risk.

To isolate the true effects of an active lifestyle, more cost-effective and accurate measures of physical activity should be implemented in our large-scale national surveys and longitudinal epidemiological studies. The National Health and Nutrition Examination Survey (5) uses a comprehensive series of questions to elicit responses about specific activities and the time spent engaging in them. These data are then converted to metabolic equivalents to calculate the energy expended. However, even surveys that collect detailed self-report measures significantly overestimate the percentage of people who meet physical activity recommendations, compared with studies that use more objective measures (10). Moreover, the most frequently cited state behavioral data set, the Behavioral Risk Factor Surveillance System, still uses a single question to assess sedentary behavior. The responses to this question only reveal the high percentage of Americans who are not engaged in physical activity for enjoyment, not their actual energy expenditure. Our most frequently used measures are insufficient to determine whether respondents are truly sedentary, irregularly active, or regularly active. Measures of physical activity must discriminate across the energy expenditure spectrum to accurately assess the dose-response effect of physical activity on morbidity and mortality rates (11). Technological advances in accelerometry and geopositioning systems are helping to unlock the door to finite objective measures of physical activity behavior and should be used if timing, resources, and participant convenience allow.

Establishing physical activity as a distinct factor in health outcomes will provide more evidence and impetus for environmental and policy changes to promote physical activity in US communities. Similarly, continued refinement of evidence-based recommendations, such as the newly released national physical activity guidelines (12), is needed. We encourage health care providers to counsel their patients to become more active as a primary and secondary preventive strategy for chronic disease, not just as a remedy for weight management.

We conclude that sufficient evidence demonstrates that the effects of weight status on cardiovascular and metabolic risk can be mediated by physical activity and CRF (5-9). As public health researchers, we should refocus our investigations to better understand the separate effects of diet and physical activity in the total population, regardless of a person's weight status, and use the most objective measures possible. These efforts will enable us to determine the most favorable physical activity and fitness levels for all Americans to reach optimal health and prevent illness.
